# Time to treat the climate and nature crisis as one indivisible global health emergency^†^

**DOI:** 10.1093/jnci/djad209

**Published:** 2023-10-25

**Authors:** Kamran Abbasi, Parveen Ali, Virginia Barbour, Thomas Benfield, Kirsten Bibbins-Domingo, Stephen Hancocks, Richard Horton, Laurie Laybourn-Langton, Robert Mash, Peush Sahni, Wadeia Mohammad Sharief, Paul Yonga, Chris Zielinski

**Affiliations:** Editor-in-Chief, *BMJ*; Editor-in-Chief, *International Nursing Review*; Editor-in-Chief, *Medical Journal of Australia*; Editor-in-Chief, *Danish Medical Journal*; Editor-in-Chief, *JAMA*; Editor-in-Chief, *British Dental Journal*; Editor-in-Chief, *The Lancet*; University of Exeter; Editor-in-Chief, *African Journal of Primary Health Care & Family Medicine*; Editor-in-Chief, *National Medical Journal of India*; Editor-in-Chief, *Dubai Medical Journal*; Editor-in-Chief, *East African Medical Journal*; University of Winchester

Over 200 health journals call on the United Nations, political leaders, and health professionals to recognise that climate change and biodiversity loss are one indivisible crisis and must be tackled together to preserve health and avoid catastrophe. This overall environmental crisis is now so severe as to be a global health emergency.

The world is currently responding to the climate crisis and the nature crisis as if they were separate challenges. This is a dangerous mistake. The 28^th^ Conference of the Parties (COP) on climate change is about to be held in Dubai while the 16^th^ COP on biodiversity is due to be held in Turkey in 2024. The research communities that provide the evidence for the two COPs are unfortunately largely separate, but they were brought together for a workshop in 2020 when they concluded that: “Only by considering climate and biodiversity as parts of the same complex problem…can solutions be developed that avoid maladaptation and maximize the beneficial outcomes” (1).

As the health world has recognised with the development of the concept of planetary health, the natural world is made up of one overall interdependent system. Damage to one subsystem can create feedback that damages another—for example, drought, wildfires, floods and the other effects of rising global temperatures destroy plant life, and lead to soil erosion and so inhibit carbon storage, which means more global warming (2). Climate change is set to overtake deforestation and other land-use change as the primary driver of nature loss (3).

Nature has a remarkable power to restore. For example, deforested land can revert to forest through natural regeneration, and marine phytoplankton, which act as natural carbon stores, turn over one billion tonnes of photosynthesising biomass every eight days (4). Indigenous land and sea management has a particularly important role to play in regeneration and continuing care (5).

Restoring one subsystem can help another—for example, replenishing soil could help remove greenhouse gases from the atmosphere on a vast scale (6). But actions that may benefit one subsystem can harm another—for example, planting forests with one type of tree can remove carbon dioxide from the air but can damage the biodiversity that is fundamental to healthy ecosystems (7).

## The impacts on health

Human health is damaged directly by both the climate crisis, as the journals have described in previous editorials (8,9), and by the nature crisis (10). This indivisible planetary crisis will have major effects on health as a result of the disruption of social and economic systems—shortages of land, shelter, food, and water, exacerbating poverty, which in turn will lead to mass migration and conflict. Rising temperatures, extreme weather events, air pollution, and the spread of infectious diseases are some of the major health threats exacerbated by climate change (11). “Without nature, we have nothing,” was UN Secretary-General António Guterres’s blunt summary at the biodiversity COP in Montreal last year (12). Even if we could keep global warming below an increase of 1.5°C over pre-industrial levels, we could still cause catastrophic harm to health by destroying nature.

Access to clean water is fundamental to human health, and yet pollution has damaged water quality, causing a rise in water-borne diseases (13). Contamination of water on land can also have far-reaching effects on distant ecosystems when that water runs off into the ocean (14). Good nutrition is underpinned by diversity in the variety of foods, but there has been a striking loss of genetic diversity in the food system. Globally, about a fifth of people rely on wild species for food and their livelihoods (15). Declines in wildlife are a major challenge for these populations, particularly in low- and middle-income countries. Fish provide more than half of dietary protein in many African, South Asian and small island nations, but ocean acidification has reduced the quality and quantity of seafood (16).

Changes in land use have forced tens of thousands of species into closer contact, increasing the exchange of pathogens and the emergence of new diseases and pandemics (17). People losing contact with the natural environment and the declining loss in biodiversity have both been linked to increases in noncommunicable, autoimmune, and inflammatory diseases and metabolic, allergic and neuropsychiatric disorders (10,18). For Indigenous people, caring for and connecting with nature is especially important for their health (19). Nature has also been an important source of medicines, and thus reduced diversity also constrains the discovery of new medicines.

Communities are healthier if they have access to high-quality green spaces that help filter air pollution, reduce air and ground temperatures, and provide opportunities for physical activity (20). Connection with nature reduces stress, loneliness and depression while promoting social interaction (21). These benefits are threatened by the continuing rise in urbanisation (22).

Finally, the health impacts of climate change and biodiversity loss will be experienced unequally between and within countries, with the most vulnerable communities often bearing the highest burden (10). Linked to this, inequality is also arguably fuelling these environmental crises. Environmental challenges and social/health inequities are challenges that share drivers and there are potential co-benefits of addressing them (10).

## A global health emergency

In December 2022 the biodiversity COP agreed on the effective conservation and management of at least 30 percent of the world’s land, coastal areas, and oceans by 2030 (23). Industrialised countries agreed to mobilise $30 billion per year to support developing nations to do so (23). These agreements echo promises made at climate COPs.

Yet many commitments made at COPs have not been met. This has allowed ecosystems to be pushed further to the brink, greatly increasing the risk of arriving at ‘tipping points’, abrupt breakdowns in the functioning of nature (2,24). If these events were to occur, the impacts on health would be globally catastrophic.

This risk, combined with the severe impacts on health already occurring, means that the World Health Organization should declare the indivisible climate and nature crisis as a global health emergency. The three pre-conditions for WHO to declare a situation to be a Public Health Emergency of International Concern (25) are that it: 1) is serious, sudden, unusual or unexpected; 2) carries implications for public health beyond the affected State's national border; and 3) may require immediate international action. Climate change would appear to fulfil all of those conditions. While the accelerating climate change and loss of biodiversity are not sudden or unexpected, they are certainly serious and unusual. Hence we call for WHO to make this declaration before or at the Seventy-seventh World Health Assembly in May 2024.

Tackling this emergency requires the COP processes to be harmonised. As a first step, the respective conventions must push for better integration of national climate plans with biodiversity equivalents (3). As the 2020 workshop that brought climate and nature scientists together concluded, “Critical leverage points include exploring alternative visions of good quality of life, rethinking consumption and waste, shifting values related to the human-nature relationship, reducing inequalities, and promoting education and learning” (1). All of these would benefit health.

Health professionals must be powerful advocates for both restoring biodiversity and tackling climate change for the good of health. Political leaders must recognise both the severe threats to health from the planetary crisis as well as the benefits that can flow to health from tackling the crisis (26). But first, we must recognise this crisis for what it is: a global health emergency.
